# Long-Term Impact of Urgent Secondary Prevention After Transient Ischemic Attack and Minor Stroke: Ten-Year Follow-Up of the EXPRESS Study

**DOI:** 10.1161/STROKEAHA.121.034279

**Published:** 2021-10-28

**Authors:** Ramon Luengo-Fernandez, Linxin Li, Louise Silver, Sergei Gutnikov, Nicola C. Beddows, Peter M. Rothwell

**Affiliations:** Wolfson Centre for the Prevention of Stroke and Dementia, Nuffield Department of Clinical Neurosciences, John Radcliffe Hospital, University of Oxford, United Kingdom.

**Keywords:** costs and cost analysis, life expectancy, maintenance, population, quality of life, secondary prevention

## Abstract

**Background and Purpose:**

Urgent assessment aimed at reducing stroke risk after transient ischemic attack or minor stroke is cost-effective over the short-term. However, it is unclear if the short-term impact is lost on long-term follow-up, with recurrent events being delayed rather than prevented. By 10-year follow-up of the EXPRESS study (Early Use of Existing Preventive Strategies for Stroke), previously showing urgent assessment reduced 90-day stroke risk by 80%, we determined whether that early benefit was still evident long-term for stroke risk, disability, and costs.

**Methods:**

EXPRESS was a prospective population-based before (phase 1: April 2002–September 2004; n=310) versus after (phase 2: October 2004–March 2007; n=281) study of the effect of early assessment and treatment of transient ischemic attack/minor stroke on early recurrent stroke risk, with an external control. This report assesses the effect on 10-year recurrent stroke risk, functional outcomes, quality-of-life, and costs.

**Results:**

A reduction in stroke risk in phase 2 was still evident at 10 years (55/23.3% versus 82/31.6%; hazard ratio=0.68 [95% CI, 0.48–0.95]; *P*=0.024), as was the impact on risk of disabling or fatal stroke (17/7.7% versus 32/13.1%; hazard ratio=0.54 [0.30–0.97]; *P*=0.036). These effects were due to maintenance of the early reduction in stroke risk, with neither additional benefit nor rebound catch-up after 90 days (post-90 days hazard ratio=0.88 [0.65–1.44], *P*=0.88; and hazard ratio=0.83 [0.42–1.65], *P*=0.59, respectively). Disability-free life expectancy was 0.59 (0.03–1.15; *P*=0.043) years higher in patients in phase 2, as was quality-adjusted life expectancy (0.49 [0.03–0.95]; *P*=0.036). Overall, 10-year costs were nonsignificantly higher in patients attending the phase 2 clinic ($1022 [-3865–5907]; *P*=0.66). The additional cost per quality-adjusted life year gained in phase 2 versus phase 1 was $2103, well below current cost-effectiveness thresholds.

**Conclusions:**

Urgent assessment and treatment of patients with transient ischemic attack or minor stroke resulted in a long-term reduction in recurrent strokes and improved outcomes, with little atrophy of the early benefit over time, representing good value for money even with a 10-year time horizon. Our results suggest that other effective acute treatments in transient ischemic attack/minor stroke in the short-term will also have the potential to have long-term benefit.

## Abstract 2

Supplemental Digital Content is available in the text.

Treatments exist to prevent recurrent stroke after transient ischemic attack (TIA) and minor stroke.^1–5^ These interventions could reduce the short-term risk of recurrent stroke by as much as 90%.^6,7^ For interventions to be most effective, however, TIA and minor stroke have to be diagnosed and treated urgently, given the high risk of recurrent stroke during the first 7 days after the event.^8–10^

In the past, in countries such as the United Kingdom, where the vast majority of TIA and minor stroke were first assessed in primary care,^11^ patients faced delays of ≈20 days between event onset and treatment prescription.^12^ The EXPRESS study (Early Use of Existing Preventive Strategies for Stroke) showed that by reducing the delays between event onset and treatment prescription to 1 day, through the set-up of urgent outpatient TIA clinics, the 90-day risk of recurrent stroke was reduced from 10.3% to 2.1% (ie, a reduction of 80%, *P*=0.0001).^12^ In addition, EXPRESS showed that these urgent clinics significantly reduced hospital care costs associated with vascular events and disability at 6 months postindex event.^13^ Similar outcomes were also achieved in the SOS-TIA study (A Transient Ischemic Attack Clinic With Round-the-Clock Access).^14^ Although these studies were nonrandomized they have been widely regarded in clinical guidelines as providing high-quality evidence of benefit of acute assessment, and the substantial reductions in early risk of recurrent stroke have since been confirmed in randomized trials of acute antiplatelet treatment. Additionally, outpatient management of minor stroke and TIA has also been implemented outside of Europe.^15^

Despite the very considerable benefits of urgent TIA/minor stroke clinics over the short-term, evidence that such acute preventative interventions impact on outcomes and costs over the long-term is limited. In the CHANCE trial (Clopidogrel in High-Risk Patients With Acute Nondisabling Cerebrovascular Events) trial, the early benefit of dual antiplatelet treatment in reducing the risk of recurrent stroke persisted at 1 year but no data are available for longer-term.^16^ The early survival benefit from 1 month of aspirin versus placebo acute treatment in myocardial infarction was maintained for at least 10 years but was based on reduced severity of the initial event rather than prevention of recurrence.^17^ If the high risk of stroke after TIA/minor stroke was transient and risk of recurrence was therefore permanently reset to a lower level urgent secondary prevention, one might expect the early benefit of acute treatment in studies such as EXPRESS or SOS-TIA to be maintained during long-term follow-up. However, if patients at high early risk continued to be at high risk of recurrent stroke in the long-term, any benefit of acute intervention might diminish with time, with events only being delayed rather than being prevented completely. Using the EXPRESS study, we assessed to what extent the short-term benefits, in terms of reduced risk of recurrent stroke, reduction in disability and hospital care costs, of urgent TIA/minor stroke clinics, were maintained over a period of 10 years.

## Methods

Requests for data will be considered by P.M. Rothwell. Health service resource use data were provided under a licence that does not permit sharing.

The EXPRESS study was a population-based sequential comparison study nested within the OXVASC (Oxford Vascular Study), the methods of which have been reported previously.^12,13,18^ At the time of the EXPRESS study, the OXVASC study population comprised about 91 000 individuals who were registered at 9 primary-care practices across Oxfordshire, United Kingdom. Registration of patients into the study began on April 1, 2002. Informed, formal, written consent was obtained from all patients included in the analyses.

Phase 1 of EXPRESS ran from April 1, 2002, to September 30, 2004, during which time primary-care physicians referred any patient who was suspected of having a TIA or nondisabling stroke to the study clinic. The OXVASC team then contacted the patient to arrange a clinic appointment as soon as possible, but often with a delay of 1 to 2 days. The patient was seen in a daily (weekdays only) hospital outpatient clinic (or at home if too frail to attend hospital), and brain imaging (usually computed tomography) and ECG were obtained on the same day. Carotid ultrasound imaging (all patients) and transthoracic or transoesophageal echocardiography (when clinically indicated) were arranged during the following week. After clinic assessment and investigation, the primary-care physician was faxed a report that included treatment recommendations. The treatment protocol recommended to the primary-care physician was tailored to the individual patient but generally included: aspirin in patients not already on antiplatelet therapy (75 mg daily), or clopidogrel if aspirin was contraindicated; simvastatin (40 mg daily); blood pressure lowering unless systolic blood pressure was below 130 mm Hg on repeated measurement (either by increases in existing medication, or by commencement of perindopril 4 mg daily with or without indapamide 1.25 mg daily); and anticoagulation as required. In patients seen within 48 hours of their event, or those seen within 7 days who were thought to be at particularly high early risk, clopidogrel (75 mg daily, to be stopped after 30 days) was recommended in addition to aspirin. Brain imaging was required before starting combination antiplatelet treatment or anticoagulation after a minor stroke. In phase 1, medication was not started in the clinic and it often took several days or weeks for prescription of treatment in primary care. In phase 1, median time from seeking medical attention to first prescription of treatments recommended in the faxed report from the study clinic was 20 (interquartile range, 8–53) days.^12^

In phase 2 (October 1, 2004, to March 31, 2007), 2 important changes were made: delays to acute assessments were reduced; and treatment was initiated immediately after assessment rather than subsequently in primary care. Primary-care physicians were requested to send all patients directly to the study clinic immediately after they presented for medical attention, with no need for a prearranged appointment, after which treatment was initiated immediately. Patients were assessed in the same way as in phase 1, but all those who were considered to have had a TIA or stroke were given aspirin 300 mg to take in the clinic, together with a prescription for a 4-week supply of any other study medication (using the same treatment protocol as in phase 1) to start on the same day. A loading dose of clopidogrel (300 mg) was also prescribed in cases in whom this drug was initiated. A computed tomography brain scan was obtained during the clinic for patients with incomplete resolution of symptoms at the time of assessment to exclude intracerebral hemorrhage before giving aspirin, clopidogrel, or anticoagulants. A report of the assessment, investigations, and treatment given was faxed to the primary-care physician as soon as possible after the clinic (usually within 24 hours). These changes reduced the median delay from seeking medical attention to first treatment to 1 (interquartile range, 0–3) day (*P*<0.0001 compared with phase 1).^12^

In both phases, the study clinician recorded detailed clinical information and the premorbid modified Rankin Scale score.^19^ As reported previously,^12^ the protocols for investigation and the treatments recommended were identical in both phases of the study, except that treatment was initiated in the study clinic in phase 2.

All patients were followed-up face-to-face by a research nurse or clinical research fellow at 1, 6, 12, 60, and 120 months after the index event, and patients were asked about any new neurological symptoms or any bleeding that required medical attention, with patients being reassessed with the modified Rankin Scale. At each follow-up, patients were asked to complete the Euroqol 5-Dimensions 3-levels questionnaire (EQ-5D-3L) to assess their health-related quality-of-life.^20^ Recurrent vascular events were also identified acutely by overlapping methods of hot and cold pursuit and assessed by a study neurologist.^18^ All potential recurrent strokes were also subsequently reviewed by the same senior neurologist (P.M. Rothwell), who was blinded to the date of the index EXPRESS event.

Although the set-up of the phase 2 EXPRESS clinic did not incur any additional costs in the research setting in which OXVASC operates, when rolled across the health service, urgent TIA/minor stroke clinics were estimated to have an additional cost.^21^ As a result, based on the results of a previous costing study,^21^ which included staffing, overheads, imaging and labs, we assumed that the costs of the phase 1 and phase 2 clinics would be $755 and $582 per patient (updated to 2017/2018 prices).

All patients then had centralized mortality and hospital resource use follow-up. Hospital care resource use and costs after assessment at one of the EXPRESS clinics were obtained over the 10-year follow-up.^11,22^ Briefly, patients’ hospital records from the Oxford University Hospitals National Health Service Foundation Trust and centrally held National Health Service Hospital Episode Statistics records were reviewed for any day case or hospitalization. Each hospitalization was valued using the 2017/2018 Health Resource Group English tariff that groups together similar clinical procedures that cost an equivalent amount to deliver. Health Resource Groups for each hospitalization, and any additional payments received for the provision of additional services, were obtained using the Health Resource Group grouper (version 4+ 2017/2018) software (National Health Service Digital, Leeds, United Kingdom). Health Resource Groups were then linked to a series of elective and emergency reference costs obtained from the 2017/2018 schedule of National Health Service reference costs.^23^ As in our previous study,^13^ the causes of admission to hospital were investigated by hot pursuit or by matching event dates to admission dates, linking of hospital information with discharge coding, and by a review of hospital notes by the OXVASC senior neurologist (P.M. Rothwell). Hospital costs were then stratified as: all-cause costs and vascular-related costs (ie, any admission within 7 days of an acute vascular event). All costs were converted from UK pound sterling to US dollars using 2018 exchange rates, adjusted for purchasing power parities (£1=$0.687; https://data.worldbank.org/indicator/PA.NUS.PPP). All outcomes and costs incurred after the first year were discounted using 3.5% annual discount rate.

### Statistical Analysis

For all analyses, the time of origin was defined as the date of the index TIA/minor stroke.

The 10-year risk of recurrent stroke, all-cause death, and disability was evaluated using Kaplan-Meier techniques, adjusted for censoring because of all-cause mortality. As with our previous study,^13^ disability was defined as modified Rankin Scale score >2. For the disability analysis, we excluded those patients disabled premorbidly. Statistically significant differences in risk between EXPRESS phases were assessed using Cox proportional hazards model. The Cox proportional hazards assumption was assessed visually.

Recurrent stroke and disability were also defined as proportions, with differences between EXPRESS phases being evaluated using χ^2^ tests. EQ-5D responses were converted into utilities using UK population tariffs developed in the 1990s,^24^ when a sample of health states was valued using the time-trade-off by 3337 members of the general public,^25^ and regression equations were fitted to obtain a tariff for all 243 possible EQ-5D health states, generating a tariff ranging from −0.59 to 1.^24^ Utilities were reported as means together with their SD. Mean differences between phases are presented together with 95% CI.

A quality-adjusted survival curve was generated by plotting, against time, the product of the mean utility at each follow-up and the probability of surviving to that follow-up. This area under the curve represents the mean quality-adjusted survival (ie, 10-year quality-adjusted life year [QALYs]).^26^ Similarly, disability-free life expectancy was estimated using the product of the proportion of patients disabled at each follow-up and the probability of surviving to that follow-up. QALYs and disability-free life expectancy were reported as means alongside 95% CI, calculated using 1000 bootstrap estimates.

For all analyses of outcome, analyses were repeated in an external group of OXVASC patients with TIA or minor stroke (National Institutes of Health Stroke Scale score <3) who were never referred to an EXPRESS clinic, mainly because they presented directly to an emergency department and were admitted to hospital for observation.

Costs were reported as means (SD), with mean differences between phases presented alongside 95% CIs. Median costs (interquartile range) are also presented with differences between phases compared using the Mann-Whitney *U* test.

Long-term cost-effectiveness of the phase 2 EXPRESS clinic, when compared with phase 1 clinic, was also evaluated. The mean difference in costs between the 2 phases, including the additional costs of the phase 2 clinic, was divided by the mean difference in QALYs, to generate an incremental cost-effectiveness ratio (the additional cost per QALY gained). Uncertainty around the incremental cost-effectiveness ratio was explored using 1000 bootstrap estimates and presented in a cost-effectiveness acceptability curve, indicating where the results fall in relation to a given cost-effectiveness threshold. An intervention was judged to be cost-effective if the incremental cost-effectiveness ratio was below £20 000 ($29 107) per QALY gained.^27^

Multiple imputation was used to impute missing utility and disability values.^28–30^ As per recommended best practice, imputation was implemented separately by allocation group (ie, EXPRESS phase).^31^ Rather than imputing missing responses for each of the 5 domains of the EQ-5D, we imputed the overall EQ-5D utility.^32^ The imputation of utilities was conducted using predictive mean matching (ie, imputes data from similar patients with complete data), whereas we used logistic regression to impute for missing values of disability. Imputation was conducted using gender, age, follow-up point, death at the next follow-up point, recurrence of stroke, stroke severity, and each of previous history of myocardial infarction, TIA, stroke, peripheral vascular disease, hypertension, angina, atrial fibrillation, smoking, diabetes, and disability. We generated 40 replacement values for each missing case, generating 40 imputed data sets. For risk of death or disability, hazard ratios were obtained using the mi est: stcox command. Differences across patient groups in terms of 10-year disability-free life expectancy and QALYs gained were obtained using ordinary least squares regression using the mi estimate reg command.

All analyses were performed using STATA MP 15 (64-bit). Statistical significance was set at *P*<0.05. The Strengthening the Reporting of Observational Studies in Epidemiology guidelines were used to report the study (Table I in the Supplemental Material).

## Results

In the whole OXVASC population, 634 patients sought medical attention after TIA or stroke in phase 1 and 644 in phase 2 (ie, 1278 first presentations). Six hundred seven presentations (285 in phase 1 and 322 in phase 2) were made directly to emergency services, usually after major stroke, or were patients who were already in hospital at the time of stroke. Six hundred twenty patients with TIA or minor stroke were referred to outpatient services, 591 (95%) directly to the study clinic (310 in phase 1 and 281 in phase 2), and are the sample in our primary analysis (Figure I in the Supplemental Material).

Baseline characteristics and risk factors of the patients are reported in the Supplemental Material (Table II in the Supplemental Material). Patients were generally similar in the 2 phases of the study, including education levels (*P*=0.39) and premorbid modified Rankin Scale scores (*P*=0.13). The median (interquartile range) National Institutes of Health Stroke Scale score for the patients with minor strokes at the time of assessment in the study clinic was 1 (0–3) in both study periods.

**Figure 1.**
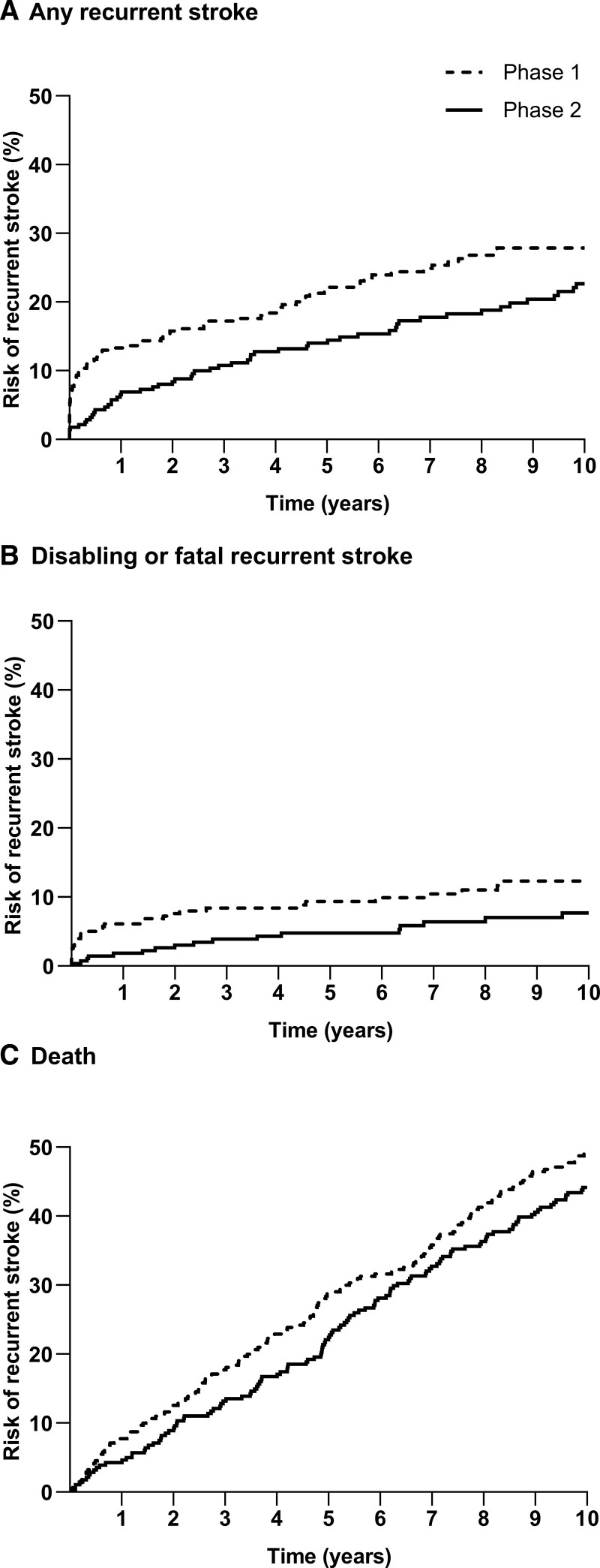
**Ten-y risk of recurrent stroke and death after seeking medical attention in all patients with transient ischemic attack (TIA) or stroke in the EXPRESS study (Early Use of Existing Preventive Strategies for Stroke) clinic cohort.** Ten-year risk of (**A**) recurrent stroke; (**B**) disabling or fatal stroke recurrent stroke; and (**C**) death in all patients with TIA or stroke in the EXPRESS study clinic cohort.

EXPRESS showed that referral to phase 2 clinic significantly reduced the number of 90-day recurrent strokes compared with referral phase 1 clinic (6 of 281 [2%] versus 32 of 310 [10%]; *P*=0.0001). At 10 years, the number of recurrent strokes continued to be significantly lower after the phase 2 versus phase 1 clinic (55 [20%] versus 82 [26%]; *P*=0.048). The actuarial 10-year risk of recurrent stroke was also significantly lower in patients attending the phase 2 clinic (hazard ratio [HR]=0.68 [95% CI, 0.48–0.95]; *P*=0.024; Figure 1, with no violation of the proportional hazards assumption). The 10-year risk of disabling/fatal recurrent stroke was also significantly lower in those patients attending the phase 2 clinic (17 [6%] versus 32 [10%]; HR=0.54 [0.30–0.97]; *P*=0.036; Figure 1, with no violation of the proportional hazards assumption). These long-term impacts on recurrent stroke risk were due solely to maintenance of the early benefit, rather than any additional benefit post-90 days. Recurrent stroke risk after 90 days did not differ in phase 2 versus phase 1 for recurrent stroke and or for disabling stroke (HR, 0.88 [0.65–1.44], *P*=0.88; and 0.83 [0.42–1.65], *P*=0.59, respectively).

At 10 years, the proportion of patients alive after referral to the EXPRESS clinic was similar in the 2 phases (phase 1: 158 of 310 [51%] versus phase 2: 158 of 281 [56%]; *P*=0.20, Table III in the Supplemental Material), with 10-year risk of death being nonsignificantly reduced in phase 2 (HR, 0.84 [0.66–1.07]; *P*=0.15; Figure 1). Ten-year discounted life expectancy was 5.58 years in patients attending phase 1 clinic versus 5.87 years in patients attending phase 2 clinic (mean difference: 0.29 [95% CI, −0.06 to 0.66]; *P*=0.08; Table).

**Table.**
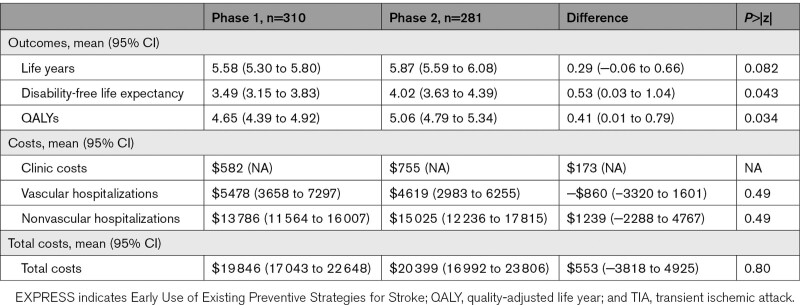
Ten-Year Discounted Outcomes and Costs After Seeking Medical Attention in All Patients With TIA or Stroke in the EXPRESS Study Clinic Cohort

The proportion of patients disabled at 10 years was lower after the phase 2 versus phase 1 clinic (30 out 158 [27%] versus 44 out of 158 survivors [36%], Table III in the Supplemental Material), with the risk of time to disability or death being significantly lower in patients attending the phase 2 (HR, 0.74 [0.60–0.91], *P*=0.004, Figure III in the Supplemental Material; with no violation of the proportional hazards assumption), a finding that remained after multiple imputation for cases in which disability information were missing (HR, 0.74 [0.60–0.90]; *P*=0.003). Again, this benefit was attributable to maintenance of the early effect of intervention, with no additional benefit after 90 days, with risk of death or disability after 90 days being similar across phases (HR, 0.94 [0.73–1.20]; *P*=0.62). Patients attending the phase 2 clinic had a significantly higher 10-year discounted disability-free life expectancy than those attending phase 1 (4.02 versus 3.49 years; mean difference: 0.53 [0.03–1.04]; *P*=0.043; Table).

Data on health-related quality-of-life (EQ-5D) at 10 years were available for 106 (67%) survivors in phase 1 and 106 (67%) in phase 2 (Table V in the Supplemental Material). At each follow-up, health-related quality-of-life was similar across the 2 groups, with utility not varying across phases (Supplemental Material). However, when utility was combined with survival, the 10-year quality-adjusted life expectancy was higher in patients attending the phase 2 clinic (5.06 versus 4.65 QALYs; *P*=0.03; Table). Therefore, over 10 years, patients attending the phase 2 clinic lived 0.41 (95% CI, 0.01–0.79) QALYs more than those attending the phase 1 clinic. In the multiple imputation analysis, patients attending the phase 2 clinic gained 0.33 (0.02–0.63; *P*=0.041) more QALYs than in the phase 1 clinic.

For our external control group, that is, patients with TIA and stroke who were not referred to a study clinic, mainly because they were seen in an emergency department and then admitted to hospital, we found no differences between phase 1 and phase 2 in recurrent stroke; and overall-, disability-free- and quality-adjusted life expectancy (Figures II and III and Tables III and IV in the Supplemental Material).

**Figure 2.**
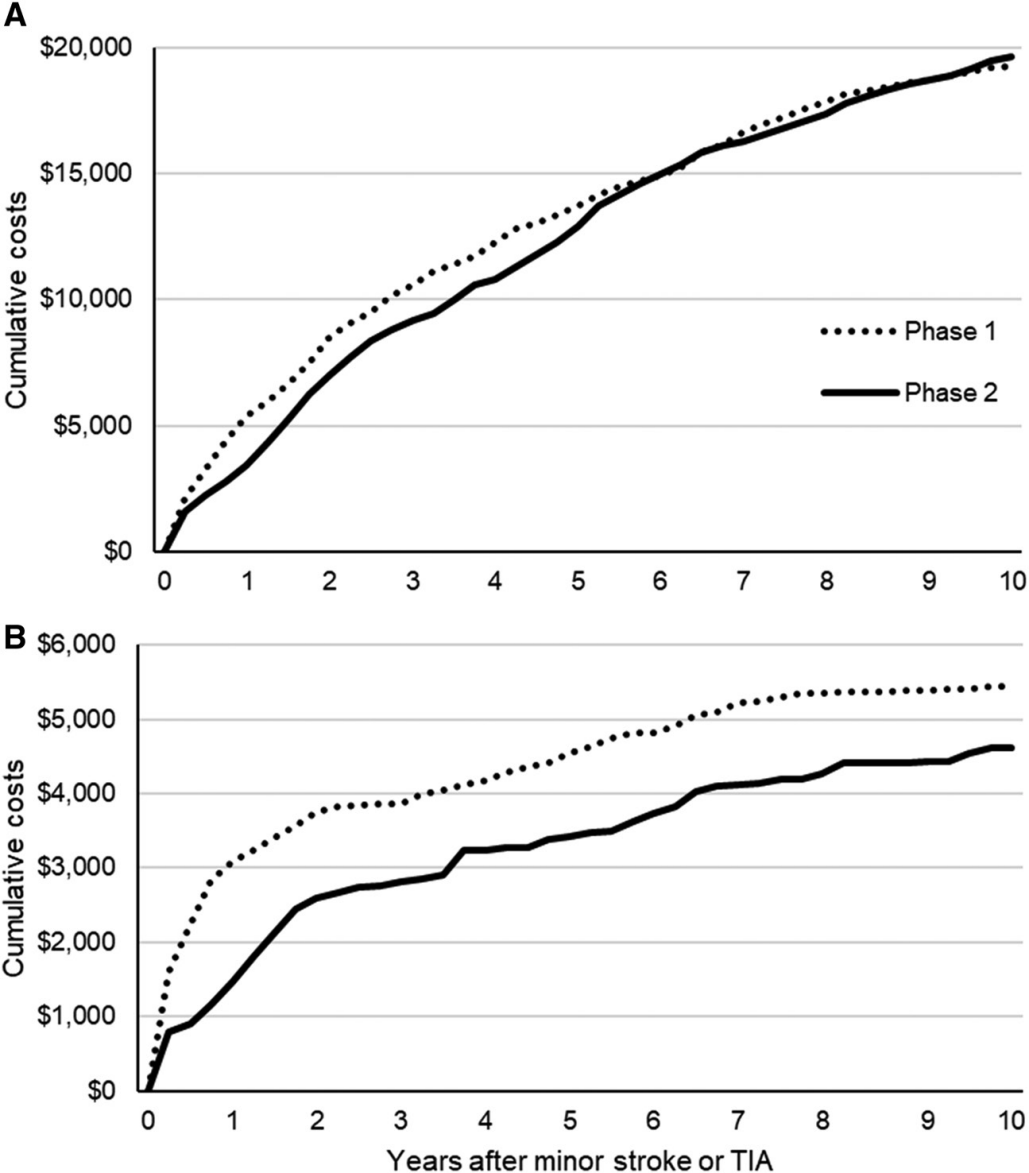
**Cumulative 10-y discounted costs in patients referred to the EXPRESS study (Early Use of Existing Preventive Strategies for Stroke) clinic after index minor stroke or transient ischemic attack (TIA).** **A**, All hospital care costs and (**B**) vascular event-related care costs.

We did not find any significant differences in overall discounted costs between patients attending the phase 1 and phase 2 clinic, over the short-term or the long-term (Figure 2). At 10 years, including the additional costs of the phase 2 clinic, overall costs were nonsignificantly higher after attending the phase 2 clinic than the phase 1 clinic ($20 399 versus $19 846; *P*=0.80). However, in terms of vascular disease costs, costs were significantly lower after attending the phase 2 clinic up until 1 year following index event ($1480 versus $3141 in phase 1; *P*=0.04). By 10 years, costs were similar across phases ($4619 in phase 2 versus $5478 in phase 1; *P*=0.49). Median discounted costs are presented in Table VI in the Supplemental Material.

**Figure 3.**
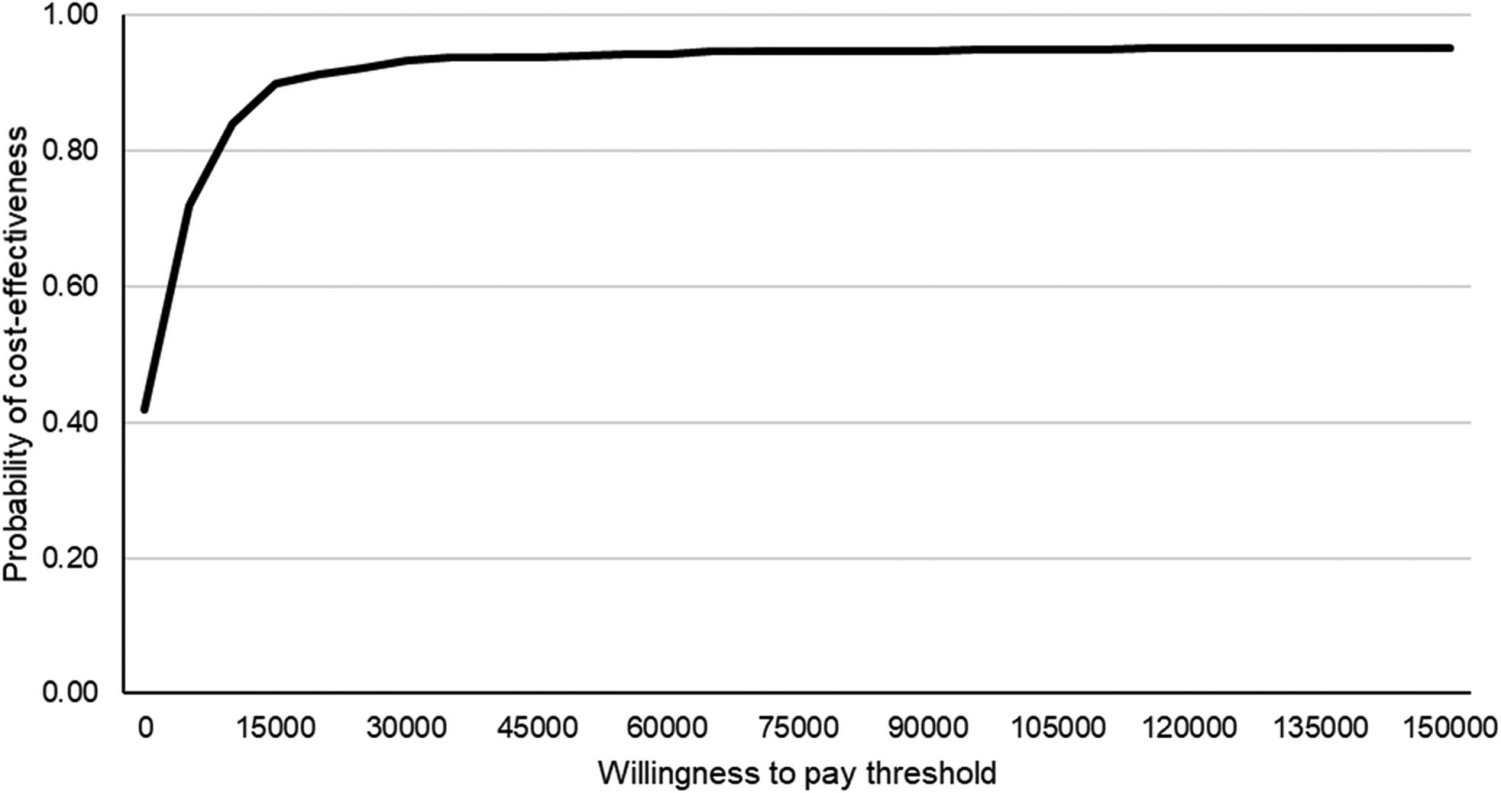
Probability of the Phase 2 EXPRESS (Early Use of Existing Preventive Strategies for Stroke) clinic being cost-effective when compared with the Phase 1 clinic at differing willingness to pay thresholds ($).

Although nonsignificant, we found that the phase 2 clinic increased overall hospital care costs at 10 years compared with the phase 1 clinic (Table). However, assessment of the cost-effectiveness of the phase 2 versus phase 1 clinic showed that the additional cost per QALY gained was $1349, resulting in a 0.931 probability of the phase 2 clinic being cost-effective, at a $29 000 per QALY threshold. This probability did not differ as the threshold increased (Figure 3).

## Discussion

Despite the considerable benefits of urgent assessment and treatment of TIA/minor stroke over the short-term,^12–14^ questions remained about long-term impact. If patients at high early risk continued to be at high risk of recurrent stroke in the long-term, any benefit of acute intervention might diminish with time, with events only being delayed by acute treatment rather than being prevented completely.

In this long-term follow-up of EXPRESS, we found that the substantial reductions in recurrent stroke at 90 days in phase 2, and the improved functional outcomes and reduced vascular costs at 6 months,^12,13^ were maintained at 10 years, including the reduction in risk of disabling/fatal recurrent stroke, resulting in an increase in the 10-year disability-free life expectancy of over half a year. We found that these long-term benefits were due to maintenance of the early effect, rather any additional benefit accrued after 90 days of the initial event, with no evidence either of rebound or loss of initial benefit. Although there were no differences in survival and quality-of-life between the 2 phases, when combined, as with 10-year disability-free life expectancy, 10-year quality-adjusted life expectancy was approximately half a year (0.41 QALYs; *P*=0.034) higher in patients attending the phase 2 clinic.

Overall, as identified in our previous study,^13^ attendance at a phase 2 clinic nonsignificantly increased overall costs over the short-term. We also found that this held over the long-term, with costs nonsignificantly higher by $553 (*P*=0.80) after attendance in the phase 2 clinic. In terms of vascular-related hospital care costs, the significant savings observed at 6 months,^13^ were carried over until 1 year, whereby vascular costs were $1661 (*P*=0.04) lower in patients attending phase 2 than phase 1 clinics. However, by 10 years, we no longer found any statistically significant differences across phases. This could be partly explained, in turn, by the trend towards higher life expectancy (0.29 years more; *P*=0.082) in phase 2, which could have resulted in patients spending more time at risk of incurring hospital costs. Despite this, however, we found that, over the long-term, urgent TIA/minor stroke outpatient clinics represent extremely good value for money (probability of being cost-effective >90%) as an effective intervention to reduce the long-term risk of stroke and disabling stroke.

To the best of our knowledge, this is the first study evaluating the 10-year risk of stroke recurrence in a TIA and minor stroke population. We found that the cumulative risk of recurrent stroke was 23.3% during the 10-year follow-up, which was in line with the TIAregistry.org project which reported a 5-year risk of 9.5%.^33^ Although we showed that the substantial benefit of the urgent interventions such as those proposed in the EXPRESS study and in the TIAregistry was maintained during the 10-year follow-up, it is worth noting that most of the recurrent strokes happened after the first 90 days (6 versus 49 in phase 2), highlighting the importance of better secondary prevention measures in reducing long-term burden of stroke.

However, our study did have some limitations in addition to being nonrandomized. External biases, such as changes in health policy or management, and ongoing improvements in life expectancy, between the 2 phases, could have, in theory, explained the improvements in outcome. However, we found no evidence of this in our external control (ie, OXVASC TIA/minor stroke patients not referred to a study clinic), with rates of stroke recurrence, and overall-, disability-free-, and quality-adjusted life expectancy all being similar across the phases. Second, EQ-5D information at 10-year follow-up was missing for 37% of cases, with patterns of missingness being similar across phases. Reasons for missing data have been reported elsewhere.^34^ In a bid to evaluate the impact of missing data on our results, we conducted multiple imputation analyses to impute for missing disability and utility values at follow-up. The results of our analyses showed that differences between phases in terms of 10-year disability-free life expectancy and QALYs gained were very similar and that significant differences remained after imputation of missing cases.

The study was based at a single-center in the United Kingdom, therefore, some of our cost results might not be directly generalizable to other geographic locations, especially in countries with nonuniversal health care systems who generally have higher health care costs.^35^ In these cases, however, we think that urgent outpatient management will represent even better value for money, as it will prevent hospitalizations that are costlier than those observed in the United Kingdom.

As with our studies in this patient population,^36^ we only included the costs of the EXPRESS clinics and costs of subsequent hospital admissions during the 10 years following initial TIA/stroke onset. Given that we found that the phase 2 clinic was associated with an increase in disability-free life expectancy, the omission of other wider costs such as those associated with informal caregiving might have resulted in our estimates of cost-effectiveness being an underestimate.

Finally, our study only included inpatient hospital care costs and the additional costs of the phase 2 clinic. We, therefore, excluded other health care costs such as those associated with primary, other specialist outpatient, and emergency care. However, these costs only represent <12% of the long-term costs after minor stroke.^11^

In conclusion, this rigorous population-based sequential comparison of all patients, irrespective of age, presenting with TIA or minor stroke has shown that urgent investigation and treatment significantly reduces the long-term risk of recurrent stroke and disabling recurrent stroke, during 10 years of follow-up, with an increase in long-term disability-free and quality-adjusted life expectancy, making urgent acute prevention highly cost-effective. More generally, our results suggest that other new acute treatment approaches in TIA and minor stroke that are effective in the short-term will also have the potential to have long-term benefit.

## Article Information

### Acknowledgments

We thank the 5 anonymous reviewers for their comments and suggestions.

### Sources of Funding

The Oxford Vascular study is funded by the National Institute for Health Research Oxford Biomedical Research Centre, Wellcome Trust, Wolfson Foundation and British Heart Foundation. Dr Li is in receipt of a fellowship award from the Medical Research Foundation.

### Disclosures

Dr Rothwell reports personal fees from Bayer and personal fees from BMS outside the submitted work.

### Supplemental Material

Supplemental Tables I–VI

Supplemental Figures I–III

## Supplementary Material


