# Supplementary material for: Long-Term Impact of Urgent Secondary Prevention After Transient Ischemic Attack and Minor Stroke: Ten-Year Follow-Up of the EXPRESS Study
Source: Stroke. 2021 Oct 28;53(2):488–96. doi: 10.1161/STROKEAHA.121.034279 (PMC8785519; doi:10.1161/STROKEAHA.121.034279)

Online supplementary file

Long-term impact of urgent secondary prevention after TIA and minor stroke: 10-year follow-up of the EXPRESS study

Ramon Luengo-Fernandez DPhil, Linxin Li DPhil, Louise Silver DPhil, Sergei Gutnikov DPhil, Nicola C Beddows, Peter M Rothwell FMedSci.

Contents:

Table I. STROBE Statement p2

Table II. Baseline characteristics for patients attending the EXPRESS clinics and external controls p4

Table III. Death, disability (mRS>2) and death or disability after seeking medical attention in all patients with TIA or stroke in the EXPRESS Study clinic cohort p5

Table IV. 10-year undiscounted life expectancy, disability-free life-expectancy and quality-adjusted life expectancy after seeking medical attention in all patients with TIA or stroke in the EXPRESS Study clinic cohort. p6

Table V. Health-related quality of life after seeking medical attention in all patients with TIA or stroke in the EXPRESS Study clinic cohort. p7

Table VI. 10-year median (IQR) discounted costs after seeking medical attention in all patients with TIA or stroke in the EXPRESS Study clinic cohort. p8

Figure I. Flow of participants through the EXPRESS study in **(A)** Phase 1, and (**B)** Phase 2 p9

Figure II. 10-year risk of recurrent stroke after seeking medical attention in all patients with TIA or stroke p10

Figure III. 10-year risk of death or disability for: **(A)** All EXPRESS clinic patients; **(B)** TIA and minor stroke patients not seen in an EXPRESS clinic p11

Table I. STROBE Statement

	Item No	Recommendation	Page No
**Title and abstract**	1	(*a*) Indicate the study's design with a commonly used term in the title or the abstract	1
		(*b*) Provide in the abstract an informative and balanced summary of what was done and what was found	2
**Introduction**			
Background/rationale	2	Explain the scientific background and rationale for the investigation being reported	3
Objectives	3	State specific objectives, including any prespecified hypotheses	3
**Methods**			
Study design	4	Present key elements of study design early in the paper	4
Setting	5	Describe the setting, locations, and relevant dates, including periods of recruitment, exposure, follow-up, and data collection	4
Participants	6	(*a*) Give the eligibility criteria, and the sources and methods of selection of participants. Describe methods of follow-up	4-5
		(*b*) For matched studies, give matching criteria and number of exposed and unexposed	n/a
Variables	7	Clearly define all outcomes, exposures, predictors, potential confounders, and effect modifiers. Give diagnostic criteria, if applicable	4-5
Data sources/ measurement	8*	For each variable of interest, give sources of data and details of methods of assessment (measurement). Describe comparability of assessment methods if there is more than one group	5
Bias	9	Describe any efforts to address potential sources of bias	6
Study size	10	Explain how the study size was arrived at	8
Quantitative variables	11	Explain how quantitative variables were handled in the analyses. If applicable, describe which groupings were chosen and why	6-7
Statistical methods	12	(*a*) Describe all statistical methods, including those used to control for confounding	6-7
		(*b*) Describe any methods used to examine subgroups and interactions	6
		(*c*) Explain how missing data were addressed	7
		(*d*) If applicable, explain how loss to follow-up was addressed	7
		(*e*) Describe any sensitivity analyses	n/a
**Results**			
Participants	13*	(a) Report numbers of individuals at each stage of study—eg numbers potentially eligible, examined for eligibility, confirmed eligible, included in the study, completing follow-up, and analysed	8
		(b) Give reasons for non-participation at each stage	Supplementary materials (SM)
		(c) Consider use of a flow diagram	SM
Descriptive data	14*	(a) Give characteristics of study participants (eg demographic, clinical, social) and information on exposures and potential confounders	SM
		(b) Indicate number of participants with missing data for each variable of interest	SM
		(c) Summarise follow-up time (eg, average and total amount)	8

Outcome data	15*	Report numbers of outcome events or summary measures over time	SM
Main results	16	(*a*) Give unadjusted estimates and, if applicable, confounder-adjusted estimates and their precision (eg, 95% confidence interval). Make clear which confounders were adjusted for and why they were included	8-9
		(*b*) Report category boundaries when continuous variables were categorized	6
		(*c*) If relevant, consider translating estimates of relative risk into absolute risk for a meaningful time period	n/a
Other analyses	17	Report other analyses done—eg analyses of subgroups and interactions, and sensitivity analyses	9
**Discussion**			
Key results	18	Summarise key results with reference to study objectives	10
Limitations	19	Discuss limitations of the study, taking into account sources of potential bias or imprecision. Discuss both direction and magnitude of any potential bias	10-11
Interpretation	20	Give a cautious overall interpretation of results considering objectives, limitations, multiplicity of analyses, results from similar studies, and other relevant evidence	11
Generalisability	21	Discuss the generalisability (external validity) of the study results	11
**Other information**			
Funding	22	Give the source of funding and the role of the funders for the present study and, if applicable, for the original study on which the present article is based	12

*Give information separately for exposed and unexposed groups.

Note: An Explanation and Elaboration article discusses each checklist item and gives methodological background and published examples of transparent reporting. The STROBE checklist is best used in conjunction with this article (freely available on the Web sites of PLoS Medicine at http://www.plosmedicine.org/, Annals of Internal Medicine at http://www.annals.org/, and Epidemiology at http://www.epidem.com/). Information on the STROBE Initiative is available at http://www.strobe-statement.org.

Table II. Baseline characteristics for patients attending the EXPRESS clinics and external controls

	Patients attending EXPRESS clinics		External controls	
	Phase 1 n=310	Phase 2 n=281	Phase 1 n=167	Phase 2 n=224
**Age (years)**				
<80	207 (67)	189 (67)	101 (60)	126 (56)
≥80	103 (33)	92 (33)	66 (40)	98 (44)
**Male sex**	141 (45)	132 (47)	88 (53)	120 (54)
**TIA**	156 (50)	160 (57)	78 (47)	104 (46)
**Hypertension**	167 (54)	164 (59)	95 (57)	123 (56)
**Diabetes**	36 (12)	37 (13)	22 (13)	24 (11)
**Previous MI**	36 (12)	29 (10)	27 (16)	24 (11)
**Previous angina**	57 (18)	42 (15)	30 (18)	23 (11)
**Previous PVD**	18 (6)	20 (7)	14 (8)	16 (7)
**Prior antiplatelet**	139 (45)	109 (39)	73 (44)	96 (43)
**Prior statin**	63 (20)	89 (32)	37 (22)	54 (25)
**Smoking**				
Current smoker	42 (14)	41 (15)	24 (14)	24 (12)
Ex-smoker	125 (40)	120 (43)	80 (48)	(96 (46)
Never	143 (46)	118 (42)	63 (38)	88 (42)

Table III. Death, disability (mRS>2) and death or disability after seeking medical attention in all patients with TIA or stroke in the EXPRESS Study clinic cohort.

	Phase 1				Phase 2			
	**Death (%)**	**Survival (%)**	**Disabled (as % of survivors)†**	**Death or disability (%)†**	**Death (%)**	**Survival (%)**	**Disabled (as % of survivors)†**	**Death or disability (%)†**
Patients referred to EXPRESS clinic								
1 month	7 (2)	303 (8)	55 (18)	62 (20)	4 (1)	277 (99)	34 (13)	38 (14)
6 months	14 (5)	296 (95)	71 (24)	85 (27)	9 (3)	272 (97)	34 (13)*	43 (15)*
1 year	24 (8)	286 (92)	60 (22)	84 (28)	12 (4)	269 (96)	36 (14)**	48 (18)**
5 years	89 (29)	221 (71)	51 (25)	140 (48)	62 (22)	219 (78)	50 (29)	112 (47)
10 years	152 (49)	158 (51)	44 (36)	196 (71)	123 (44)	158 (56)	30 (27)	153 (65)
External controls (TIA and minor stroke patients not referred to EXPRESS clinic)								
1 month	14 (8)	153 (92)	47 (33)	61 (39)	15 (7)	209 (93)	58 (32)	73 (37)
6 months	16 (10)	151 (90)	47 (34)	63 (40)	23 (10)	201 (90)	50 (29)	73 (38)
1 year	26 (16)	141 (84)	43 (33)	69 (44)	32 (14)	192 (86)	47 (29)	79 (41)
5 years	77 (46)	90 (54)	27 (34)	104 (67)	89 (40)	135 (60)	37 (32)	125 (61)
10 years	109 (65)	58 (35)	9 (21)	117 (77)	132 (59)	92 (41)	22 (35)	153 (79)

Statistically significant at: *$p<0.001$ & ** $p<0.050$ when compared to phase 1

Table IV. 10-year undiscounted life expectancy, disability-free life-expectancy and quality-adjusted life expectancy after seeking medical attention in all patients with TIA or stroke in the EXPRESS Study clinic cohort.

	Phase 1	Phase 2	Difference	p>|z|
	Patients referred to EXPRESS clinic			
	n=310	n=281		
**Life-years**	7.3 (7.0 to 7.6)	7.7 (7.4 to 8.0)	0.39 (-0.05 to 0.83)	0.073
**Disability-free life-expectancy**	4.9 (4.5 to 5.2)	5.5 (5.0 to 5.9)	0.59 (0.03 to 1.15)	0.043
**QALYs**	5.4 (5.1 to 5.7)	5.9 (5.5 to 6.2)	0.49 (0.03 to 0.95)	0.036
	External controls (TIA and minor stroke patients not referred to EXPRESS clinic)			
	n=167	n=224		
**Life-years**	5.9 (5.4 to 6.4)	6.4 (6.0 to 6.8)	0.49 (-0.15 to 1.14)	0.101
**Disability-free life-expectancy**	3.6 (3.0 to 4.1)	3.8 (3.3 to 4.2)	0.16 (-0.52 to 0.85)	0.354
**QALYs**	4.4 (4.0 to 4.8)	4.7 (4.4 to 5.1)	0.35 (-0.22 to 0.91)	0.153

Table V. Health-related quality of life after seeking medical attention in all patients with TIA or stroke in the EXPRESS Study clinic cohort.

	Phase 1	Phase 2	Difference	p>|z|*
Patients referred to EXPRESS clinic				
1 month	0.751 (0.268) n=233	0.784 (0.227) n=207	0.032 (-0.014 to 0.078)	0.086
1 year	0.759 (0.245) n=238	0.765 (0.264) n=173	0.005 (-0.045 to -0.055)	0.421
5 years	0.759 (0.262) n=162	0.757 (0.269) n=155	-0.002 (-0.061 to 0.056)	0.530
10 years	0.705 (0.325) n=106	0.718 (0.320) n=106	0.014 (-0.074 to 0.101)	0.380
External controls (TIA and minor stroke patients not referred to EXPRESS clinic)				
1 month	0.689 (0.279) n=81	0.750 (0.249) n=122	0.061 (-0.012 to 0.135)	0.052
1 year	0.736 (0.247) n=93	0.721 (0.271) n=111	-0.015 (-0.087 to 0.057)	0.658
5 years	0.754 (0.239) n=57	0.719 (0.283) n=83	-0.035 (-0.126 to 0.055)	0.779
10 years	0.766 (0.307) n=41	0.746 (0.271) n=57	-0.020 (-0.136 to 0.097)	0.631

Table VI. 10-year median (IQR) discounted costs after seeking medical attention in all patients with TIA or stroke in the EXPRESS Study clinic cohort

	Phase 1 n=310	Phase 2 n=281	p>|z|
Clinic costs	$582 (n/a)	$755 (n/a)	n/a
Vascular hospitalisation	$0 (0-2,622)	$0 (0-2,256)	0.88
Non-vascular hospitalisations	$6,119 (1,198-18,092)	$6,055 (1,444-17,092)	0.80
Total costs	$10,214 (2,944-26,992)	$9,517 (3,458-24,070)	0.85

Figure I. Flow of participants through the EXPRESS study in **(A)** Phase 1, and **(B)** Phase 2

A

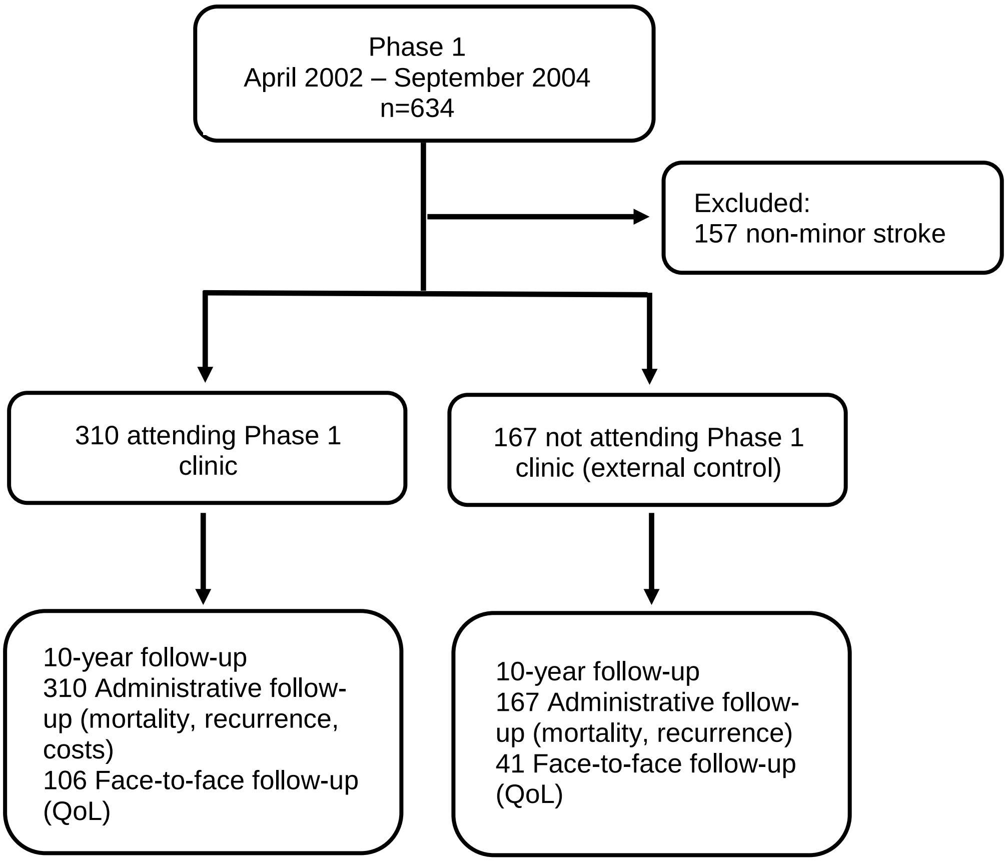

B

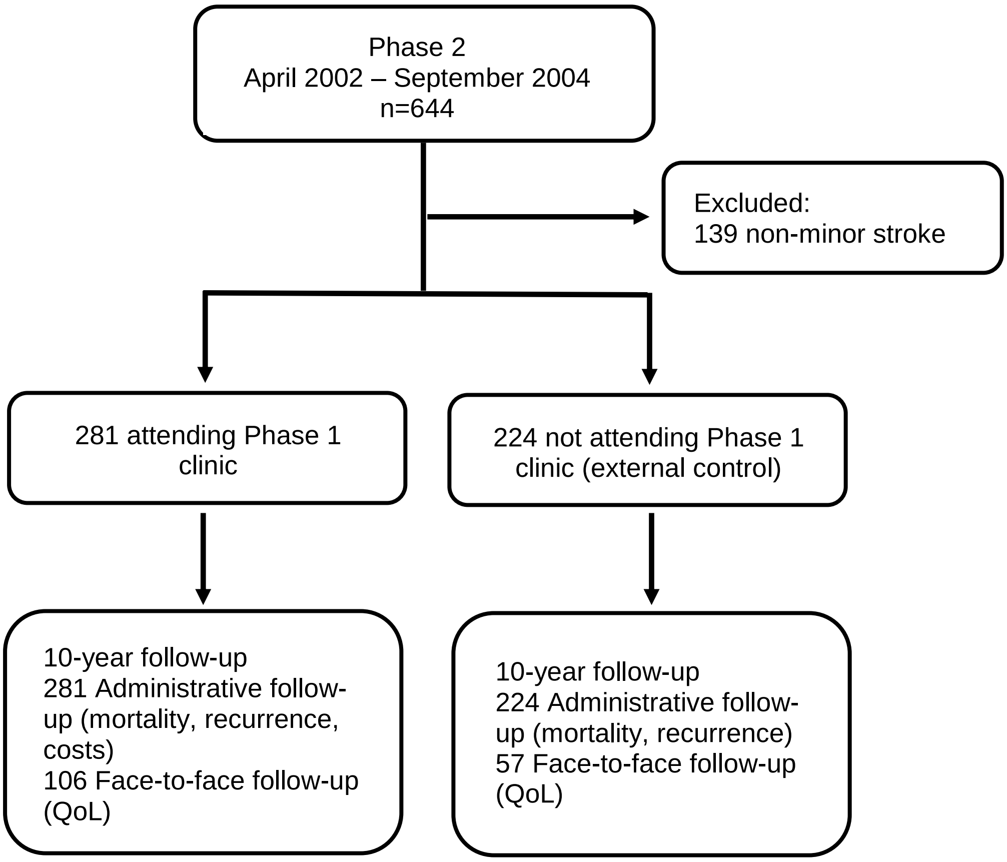

Figure II. 10-year risk of recurrent stroke after seeking medical attention in all patients with TIA or stroke

TIA and minor stroke patients not seen in the EXPRESS clinic | **All EXPRESS clinic patients**

A. Recurrent stroke

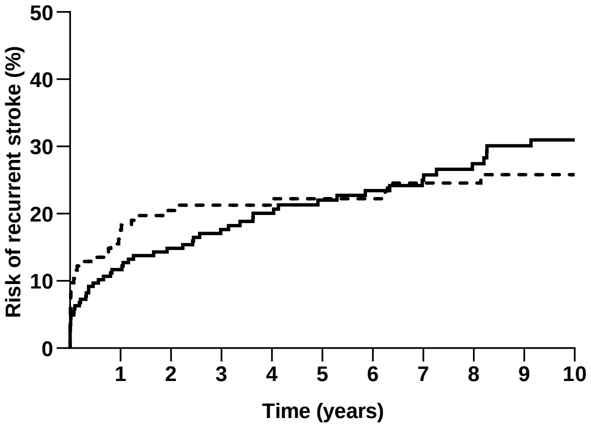

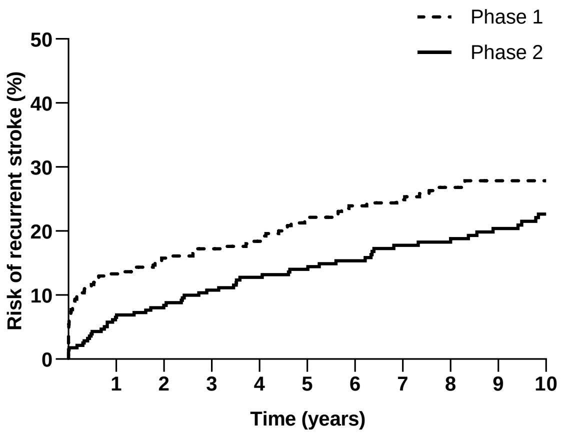

B. Recurrent disabling/fatal stroke

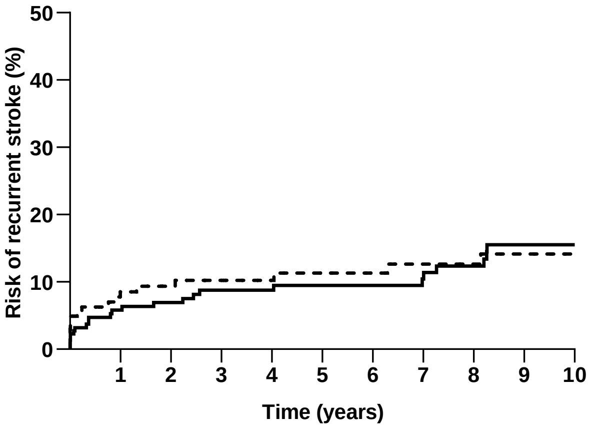

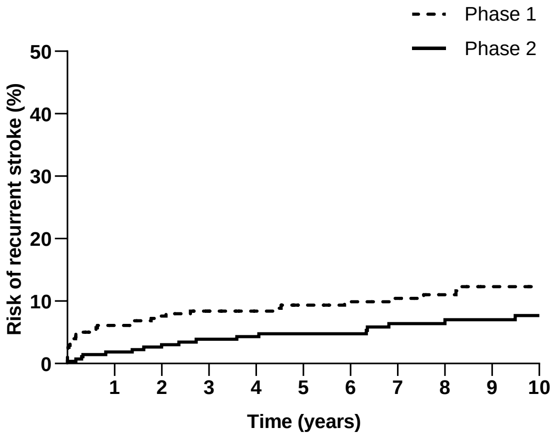

All follow-up	Phase 1	Phase 2	Log rank p
**Recurrent stroke**			
Non-EXPRESS	39/167	55/224	0.95
EXPRESS	82/310	55/281	0.024
**Recurrent disabling/fatal stroke**			
Non-EXPRESS	20/167	24/224	0.59
EXPRESS	32/310	17/281	0.036

Figure III. 10-year risk of death or disability for: **(A)** All EXPRESS clinic patients; **(B)** TIA and minor stroke patients not seen in an EXPRESS clinic

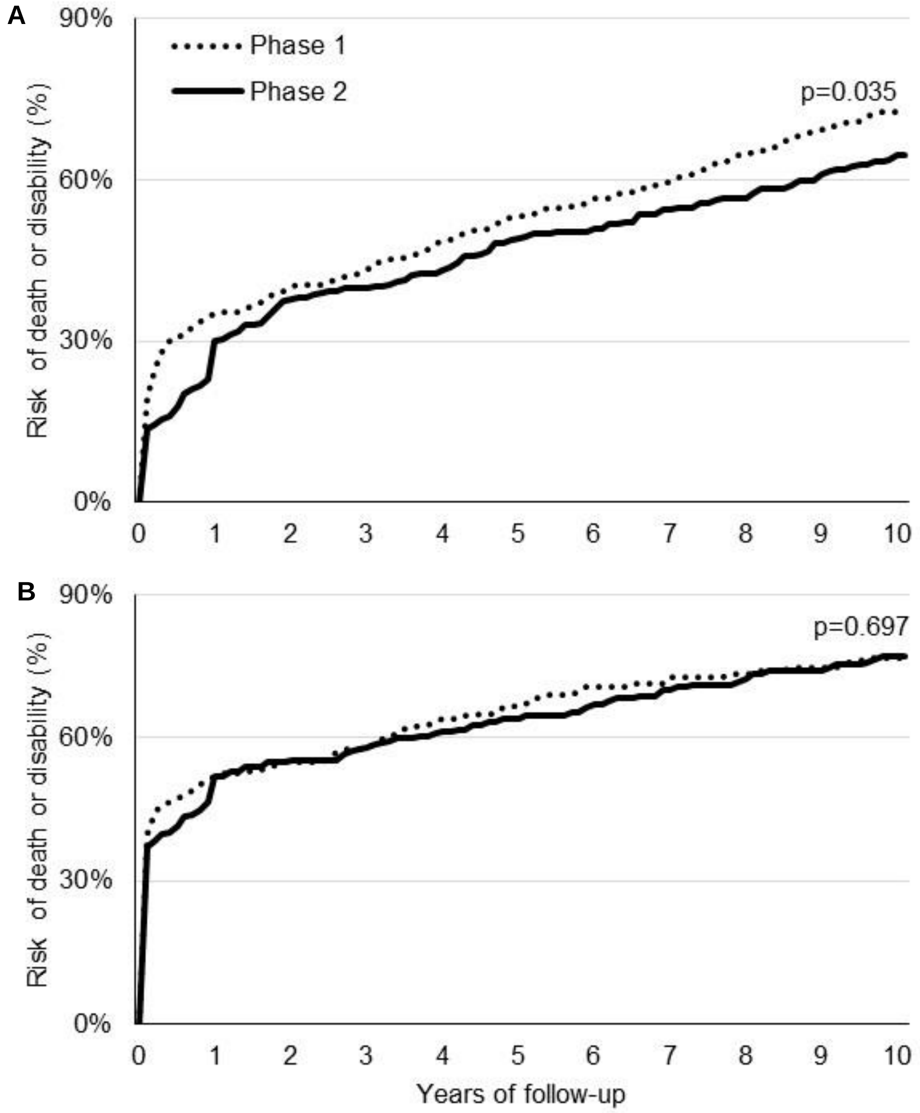

Supplement: Supplementary file 1 [file str-53-488-s001.pdf]
