# Supplementary figures and images for: Long-Term Impact of Urgent Secondary Prevention After Transient Ischemic Attack and Minor Stroke: Ten-Year Follow-Up of the EXPRESS Study
Source: Stroke. 2021 Oct 28;53(2):488–96. doi: 10.1161/STROKEAHA.121.034279 (PMC8785519; doi:10.1161/STROKEAHA.121.034279)

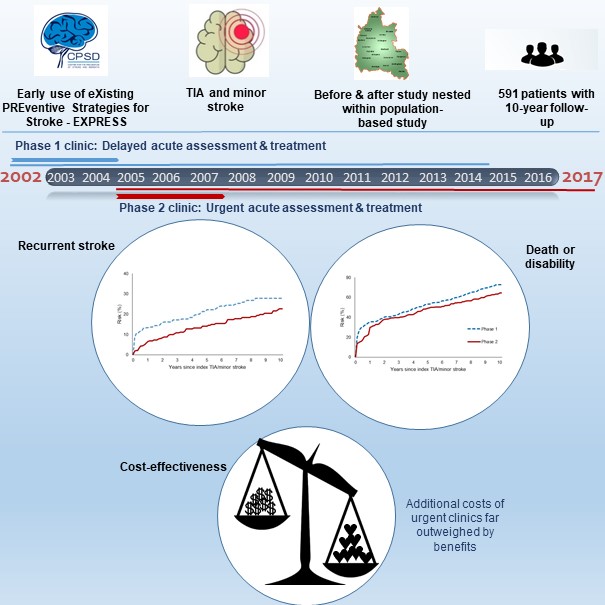

Supplement: Supplementary file 2 [file str-53-488-s002.jpg]
